# Experimental study on improving the durability of underground/underwater structures by mixing impregnating agents into unhardened concrete

**DOI:** 10.1038/s41598-025-16991-6

**Published:** 2025-09-01

**Authors:** Tetsuya Kouno, Yu Qiu, Toshiya Ichihashi

**Affiliations:** 1 Academic Assembly Faculty of Sustainable Design, University of Toyama, 3190 Gofuku, Toyama, Toyama Prefecture 930-8555 Japan; 2 University of Toyama Graduate School of Science and Engineering, 3190 Gofuku, Toyama, Toyama Prefecture 930-8555 Japan; 3 CTI Engineering Co., Ltd. (At the time of the study: University of Toyama Graduate School of Science and Engineering), Nagoya, Japan

**Keywords:** Sodium silicate-based impregnating agent, Flowability, Durability, Mechanical properties, Engineering, Environmental sciences, Materials science

## Abstract

Components exposed to soil and water are difficult to maintain during their service life, and have traditionally been designed to ensure greater durability than components exposed to air. However, in recent years, severe damage caused by aging has also been observed in components exposed to soil and water, prompting the development of methods to achieve higher durability. To address this issue, the authors developed a method (mixing method) that enhances the durability of concrete components by mixing sodium silicate-based impregnation materials with fresh concrete. In this study, the flowability, deterioration inhibition effects, and mechanical properties of concrete prepared using the mixing method were experimentally confirmed. The results demonstrated that by controlling the addition of the impregnation material, it is possible to achieve deterioration suppression effects equivalent to or superior to those of conventional methods without significantly reducing the workability or mechanical properties. In addition, an equation for estimating the mechanical properties expressed from the amount of admixture was proposed. Based on the results of this study, it is considered that the mixing method can be applied as a method for maintaining and improving the durability of concrete structures. In order to put this into practical use, it is necessary to conduct experiments under a wider range of conditions to improve the accuracy of estimating the deterioration suppression effect and the characteristics that appear, and to expand the range of applications.

## Introduction

Various measures have been taken to prevent the deterioration of concrete structures during their service life. As with bridge girders, durability can be ensured during the service life by implementing maintenance measures such as surface impregnation methods for components exposed to the air. On the other hand, maintenance activities, such as inspection and repair, are difficult to perform for underground or underwater structures during their service life. Therefore, measures are taken during the construction stage to ensure that the structures have higher durability than above-ground structures so that they can maintain the specified durability at the end of their service life without requiring repairs or maintenance during their service life. For example, the Specifications for Highway Bridges (design standard for Japanese road bridges), Eurocode (technical standard for European civil engineering structures), and AASHTO LRFD (bridge design standard for the United States) stipulate that the cover thickness of reinforced concrete structures buried in soil should be approximately 20 mm greater than that of aboveground reinforced concrete structures^1–3^. In addition, blast furnace cement and methods that improve the density of concrete by setting the water–cement ratio to a lower value than that of aboveground structures are also commonly adopted^1^.

**Fig. 1:**
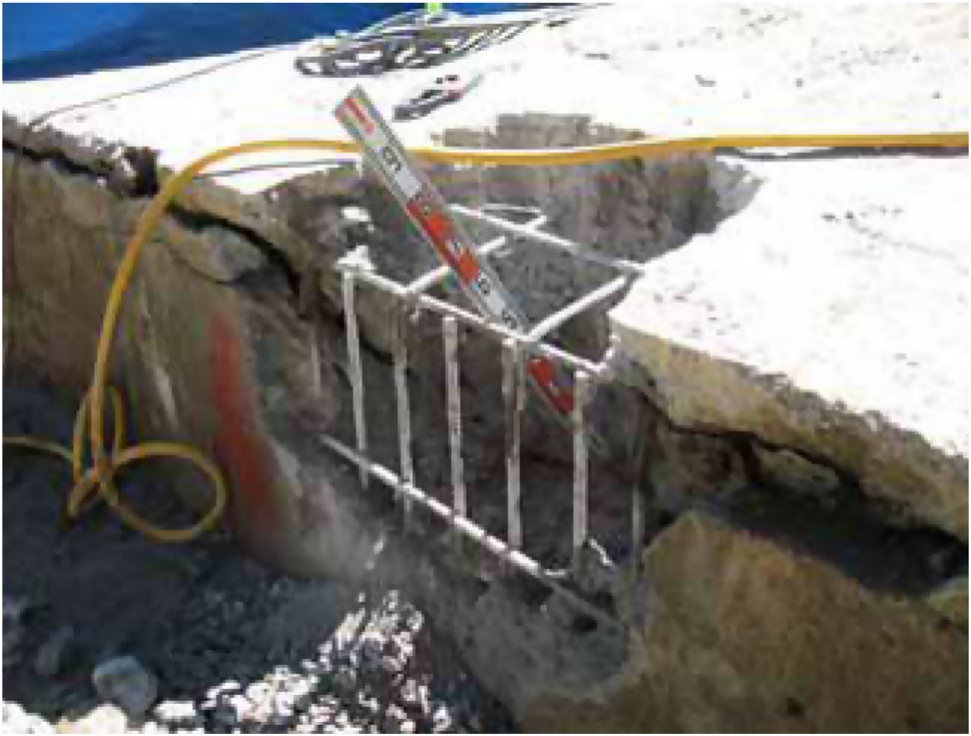
Underground structure exhibiting rebar damage due to alkali–silica reaction^5^.

However, in recent years, cases of deterioration over time owing to carbonation^4^ and alkali–silica reaction^5,6^ in underground or underwater concrete structures, such as bridge foundations and culverts, have been reported. Serious damage was reported in some of these cases, including rebar fractures, as shown in (Fig. 1). Such damage cases suggest that damage cannot be inhibited by conventional measures, such as increasing the cover thickness, and that it is necessary to provide greater durability at the time of construction than that considered sufficient in the past.

To achieve higher durability than before, methods such as further reducing the water-cement ratio (W/C) or increasing the cover thickness may be considered. However, excessive reduction of the W/C ratio can alter the load-bearing ratio between the reinforcing bars and the concrete, leading to a decrease in ductility and potentially resulting in brittle failure. Similarly, excessive increase in cover thickness can cause concrete crushing to dominate, also leading to brittle failure. To enhance durability, in addition to reducing the W/C ratio and ensuring adequate cover, new durability-enhancing measures are necessary.

In recent years, increasing attention has been given to methods of enhancing the durability of concrete by incorporating water glass into fresh mixtures. Water glass is highly alkaline and functions as an activator, promoting the dissolution of Si and Al species from calcium ions in cement, as well as from fly ash and ground granulated blast-furnace slag^7–9^. Furthermore, sodium silicate (Na₂SiO₃), the main component of water glass, reacts with Ca^2^⁺ to form calcium silicate hydrate (C–S–H) gel, thereby increasing the matrix density and reducing porosity (Eq. (1))^7,10–13^.

1 $${\text{Na}}_{{2}} {\text{SiO}}_{{3}} + {\text{Ca}}\left( {{\text{OH}}} \right)_{{2}} + {\text{H}}_{{2}} {\text{O}} \rightleftharpoons ({\text{CaO}} \cdot {\text{SiO}}_{{2}} ) \cdot {\text{H}}_{{2}} {\text{O}} + {\text{Na}}_{{2}} {\text{O}}$$

These effects contribute to improvements in early strength and durability, slowing down the progression of concrete deterioration and potentially reducing maintenance effort and cost^7^.

However, since these reactions occur during mixing, a tendency for workability loss has been observed^8–10^. In addition, due to the rapid acceleration of hydration, unhydrated cement may remain, resulting in a decrease in long-term strength^7,9,14^. These issues stem from the concentration of water glass reactions at the early stage of mixing. To address this, several studies have investigated encapsulating water glass to delay its initial reaction, allowing it to be activated only after cracks form in the concrete^14^. Nevertheless, the rupture of capsules during mixing can prevent the intended effect, and non-uniform dispersion or inconsistent rupture locations of the capsules can lead to heterogeneous strength distribution within the element^7,14,15^. Furthermore, significant variability in the resulting durability and mechanical performance remains a challenge. A review of the experimental results reported by Szewczenko et al., Cavusoglu et al., Hamsashree et al., and Ghanim et al. shows that the compressive strength reduction ratio—defined as the ratio of the compressive strength of specimens with water glass to those without—ranges from approximately 5 to 20%. Such a wide variability makes direct application to real structures difficult^7,9–11^.

Therefore, the authors considered using a sodium silicate-based impregnating agent instead of water glass. Sodium silicate-based impregnation materials have the same composition as water glass but contain less Na₂O. Therefore, while the same durability improvement mechanism as water glass is expected, the slower reaction of the impregnation material is anticipated to suppress reductions in strength and flowability, as well as variations in performance characteristics. Furthermore, the presence of unreacted components within the concrete is expected to enhance long-term density and self-healing functionality.

Based on the discussion above, this study aimed to evaluate various properties (workability, deterioration inhibition effect, and mechanical properties) of fresh concrete mixed with sodium silicate-based impregnating agents. These properties are expected to vary depending on conditions such as the concrete mix design, impregnation agent content, and time of addition of the impregnation agent. Therefore, various experiments were conducted by varying these conditions. The experiments performed included slump and slump flow tests to confirm the workability, neutralization tests to confirm the deterioration inhibition effects, and compressive strength tests to confirm the mechanical properties. As an accelerated deterioration test, neutralization testing was selected for this study because it reflects the actual deterioration phenomena occurring in underground structures and is considered to be less susceptible to factors other than the admixture of impregnating agents.

The experimental results were organized, and the changes in various properties were analyzed based on the values under each condition. In particular, a quantitative relationship between the specific condition values and compressive strength was identified, and an estimation formula for the compressive strength in the mixing method was proposed. Further, it was demonstrated that both flowability and mechanical properties could be preserved while achieving deterioration-inhibiting effects, thereby confirming the usefulness of the mixing method.

## Experimental overview

### Experimental cases

Table 1 lists the experimental cases and summarizes the specimen compositions and parameters that were changed in each test. Since there are no previous examples of mixing sodium silicate-based impregnating agents into fresh concrete, the composition and mixing ratio were determined through trial and error, including preliminary experiments, as shown below. Series A comprised preliminary experiments conducted prior to all the other experiments to confirm the feasibility of the mixing method. In this series, only a slump test was conducted in which the quantity of the impregnation material used for mixing was set as a parameter to observe the changes in fluidity and workability.

**Table 1:** Mixing proportions and experimental cases.

Experimental case		W/C	Unit weight [kg/m^3^]						Application of impregnation material		Tests		
Series	Case		Water	Cement	Fine aggregate	Coarse aggregate	Impregnation material	AE water reducing agent^a^	Application method	Timing of mixing impregnation material	Slump test\Slump flow test^b^	Carbonation test	Compressive strength test
A	CA	0.56	180	320	791	983	0	3.2	No material mixed	–	Slump test only	–	–
	MA17						17		Mixing method	Same time	Slump test only	–	–
	MA35						35		Mixing method	Same time	Slump test only	–	–
	MA156						156		Mixing method	Same time	Slump test only	–	–
I	C	0.56	177	320	807	931	0	3.2	No material mixed	–	Yes	Yes	Yes
	S						8		Surface impregnation	–	–	Yes	–
	M8						8		Mixing method	Same time	Yes	Yes	Yes
	M16						16			Same time	Yes	Yes	Yes
	M48						48			Same time	Yes	Yes	Yes
	M80						80			Same time	Yes	Yes	Yes
	M16–W45	0.45		400	897	778	16	4.0		Same time	Yes	Yes	Yes
	M16–W50	0.50		360	913	793	16	3.6		Same time			Yes
	M16–W60	0.60		300	939	815	16	3.0		Same time	Yes	Yes	Yes
II	C ‘	0.56		320	965	983	0	3.2	No material mixed	–	–	–	Yes
	M’8–m0						8		Mixing method	Same time	–	–	Yes
	M’16–m0						16			Same time	–	–	Yes
	M’16–m15						16			15 min later	–	–	Yes
	M’16–m30						16			30 min later	–	–	Yes
	M’16–m60						16			60 min later	–	–	Yes
	M’48–m0						48			Same time	–	–	Yes
	M’80–m0						80			Same time	–	–	Yes

*AE* air entraining.

^a^Series I used a high-performance AE water reducer; Series A and II used AE water reducers.

^b^Only slump test was conducted in Series A.

Based on the results of Series A, we conducted additional experiments under various conditions, including the application of the mixing methods, amount of impregnation material, water–cement ratio, and time of adding the impregnation material in the mixing method. In Series I, the parameters included the mixing amount of the impregnation material and water–cement ratio, whereas the parameters of Series II included the mixing amount and time of addition of the impregnation material. In addition to the mixing method, for comparison with the results of Series I and II, we conducted experiments involving the surface impregnation method, a commonly method for preventing concrete deterioration with using sodium silicate-based impregnating agents, and a case in which no impregnation material was mixed. The cases with the letter C in the case name are those in which no impregnation material was mixed, whereas S and M denote the cases where the experiments were conducted using the surface impregnation method and mixing method, respectively. The number following M indicates the amount of impregnation material mixed.

The amount of impregnation material in each case was determined by referring to the amount applied in the standard surface impregnation method, which is often specified as the mass of impregnation material per unit area. According to JSCE-K572-2012, the impregnation material should be applied to the side surface that is in contact with the formwork^16^. Therefore, for Case S, the impregnation material was applied to an area of 0.02 m^2^ (0.01 m^2^ per surface, two surfaces in contact with the formwork), which is the area that was in contact with the longitudinal side surface of the formwork in the specimens used in the carbonation test described below. The amount of impregnation material applied was defined for each case; because the standard amount of application of the impregnation material used in this experiment was 400 g/m^2^, 8 kg/m^3^ was applied in Case S. Therefore, the amount of impregnation material mixed in the mixing method was set to the following values: 8 kg/m^3^, which was the same as the surface application amount; 16 kg/m^3^, which was twice the surface application amount; 48 kg/m^3^, which was six times the surface application amount; and 80 kg/m^3^, which was ten times the surface application amount. The number after W in the case name is the water–cement ratio. In this study, four ratios were used: 45, 50, 56, and 60%. This value is based on the composition of the substructure and foundation of road bridges in Japan^1^. The W/C ratio for the cases without W in the name was 56%.

As mentioned above, in the mixing method, the hydration reactions of the cement and impregnation material proceed simultaneously, and the reaction of the impregnation material may inhibit the hydration reaction. We believe that the effect of the reaction of the impregnation material on the hydration reaction depends on the time of mixing the impregnation material. Hence, we also investigated cases in which the time of mixing the impregnation material was used as a parameter. In this case, when the mixing amount in Series II was 16 kg/m^3^, four timings were set: immediately after the start of mixing (M'16-m0) and 15, 30, and 60 min after mixing (M'16-m15, M'16-m30, and M’16-m60). Table 1 summarizes the tests conducted in this study.

The surface impregnation method involves applying an impregnating agent after concrete has hardened; therefore, the properties of fresh concrete remain identical to those of normal concrete. Consequently, since the slump and slump flow of Case S are considered to be the same as those of Case C, slump and slump flow tests were not performed for Case S. In addition, because it has been clarified that the change in mechanical properties due to the application of an impregnation material to the surface is extremely small^17,18^, compressive strength tests were not performed for Case S.

### Materials used

**Fig. 2:**
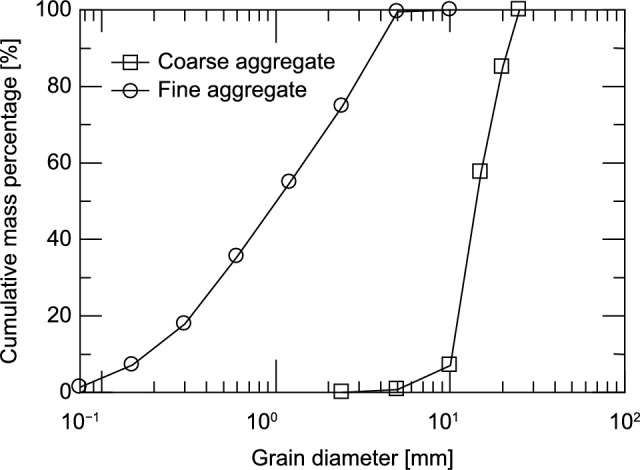
Particle size accumulation curve.

The impregnation material used in this experiment was a commercially available sodium silicate-based material. The reaction mechanism is shown in Eq. (1). The cement used was ordinary portland cement (density: 3.16 g/cm^3^). The Used aggregates were river sand (surface dry density: 2.65 g/cm^3^) as the fine aggregate, river gravel (surface dry density: 2.50 g/cm^3^) as the coarse aggregate. A high-performance air-entraining (AE) water-reducing admixture is used in Series A and I, and an AE water-reducing admixture is used in Series II. The properties of the aggregates and cement used are listed in Table 2, and the particle size accumulation curves of the aggregates are shown in (Fig. 2).

**Table 2:** List of properties of materials used in this study.

Fine aggregate		Coarse aggregate		Cement
Density [g/cm^3^]	Average particle size [mm]	Density [g/cm^3^]	Average particle size [mm]	Density [g/cm^3^]
2.65	1.05	2.50	15.02	3.16

### Slump test/slump flow test

The slump test was performed according to JIS A 1101:2020, and the slump flow test was performed only for Series I specimens, according to JIS A 1150:2020^19,20^. The slump flow was measured immediately after the slump value.

In the case of the mixing method, the reaction of the impregnation material progresses within the fresh concrete, which can cause a decrease in the slump and slump flow within a short period. Accordingly, in this study, the tests were performed several times: twice for Series A—once immediately after pouring the concrete and mixing the impregnation material, and the other 10 min after pouring the concrete and mixing the impregnation material—and three times for Series I—immediately after, 10 min after, and 40 min after pouring the concrete and mixing the impregnation material. Mixing was continued manually from the pouring of the concrete to the mixing of the impregnation material in the slump test.

### Compressive strength test

Compressive strength tests were conducted according to the JIS A 1108:2018 standard^21^. The specimens were cylindrical with a diameter of 10 cm and height of 20 cm. Three specimens were prepared for each case.

The specimens were removed 24 h after casting and cured underwater at 20 °C until the compressive strength test was performed. Generally, the compressive strength tests for concrete using ordinary portland cement are conducted at a concrete age of 28 d. However, as mentioned at the beginning, in the case of the mixing method, the hardening/setting reactions and reaction with the impregnation material proceed simultaneously. This significantly reduces the strength and rigidity at a young concrete age, which may affect the safety during casting. Therefore, in this study, in addition to the concrete age of 28 d for which the general design standard strength is set, tests were conducted for the concrete ages of 7, 14, and 21 days. Before each compressive strength test, both end faces were polished, and their dimensions were measured.

### Accelerated carbonation test

The specimens used in the accelerated carbonation test were cubes with sides of 100 mm each. The specimens were removed from the formwork 24 h after pouring and then cured underwater at 20 °C for 28 days.

Subsequently, accelerated carbonation tests were conducted according to JIS A 1153:2012 at a temperature of 20 °C, humidity of 60%, and carbon dioxide concentration of 5%^22^. In Case S, after the underwater curing period, the impregnation material was applied to the specified areas before conducting the accelerated carbonation test.

**Fig. 3:**
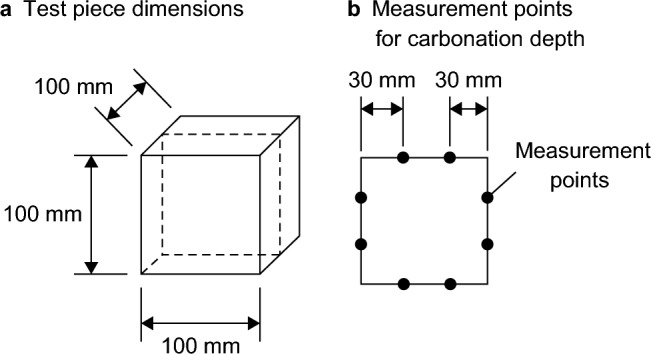
Test piece dimensions and measurement points for accelerated carbonation test.

After a specified period of time had elapsed in the accelerated environment, the specimens were cut in half using a concrete cutter (Fig. 3a), and phenolphthalein solution was sprayed onto the cut surfaces to measure the carbonation depth. The carbonation depth of each cross section was measured at a total of eight points, 30 mm from each corner, as shown in (Fig. 3b), and the distance from the concrete surface to the reddish-purple colored part was measured as the carbonation depth in 0.5 mm increments. Three specimens were used per case, and each test piece had two measurement cross sections with eight measurement points each, giving a total of 48 measurement points per case. The results for each case presented in the text are the averages of results from the 48 points.

## Test results

### Slump test/slump flow test

**Fig. 4:**
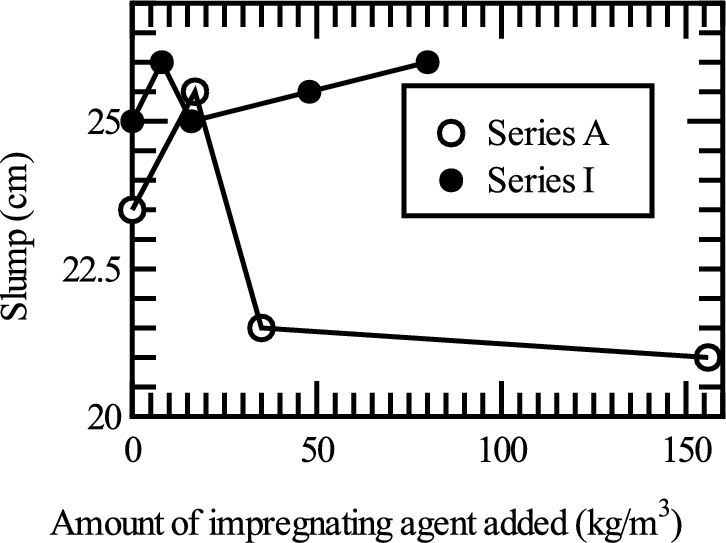
Slump test results immediately after impregnation material addition.

Table 3 shows the results of the slump and slump flow tests and Fig. 4 shows the relationship between the slump value obtained from the slump test (vertical axis) and the amount of impregnation material mixed (horizontal axis). For Series A and I, the test results are shown for cases in which the impregnation material was added immediately after mixing.

**Table 3:** Results of the slump and slump flow tests.

Series	Case	Amount of impregnation agent added [kg/m^3^]	Time from mixing to adding and testing [min]		
			0	10	40
(a) Slump test [cm]					
A	CA	0	23.5	24.0	|
	MA17	17	25.5	24.0	|
	MA35	35	21.5	18.0	|
	MA156	156	21.0	5.5	|
I	C	0	25.0	24.5	25.5
	M8	8	26.0	26.0	25.0
	M16	16	25.0	25.5	25.5
	M48	48	25.5	25.5	24.0
	M80	80	26.0	25.0	25.5

Time from mixing to adding and testing [min]	Case/amount of impregnation agent added [kg/m^3^]				
	C / 0	M8 / 8	M16 / 16	M48 / 48	M80 / 80
(b) Slump flow test [cm] (Series I)					
0	55.5	56.8	59.75	42.5	45.25
10	56.5	54.3	52.75	36.25	29
40	41.5	41.5	42.75	35.25	32.75

Figure 4 shows that the slump value for Series A decreases sharply when the mixing amount exceeds 35 kg/m^3^. The reason for this is inferred to be the increase in the amount of CSH gel produced with increase in the amount of impregnation material mixed, resulting in pseudo-hardening, as reported in the previous studies where water glass was mixed^23,24^.

**Fig. 5:**
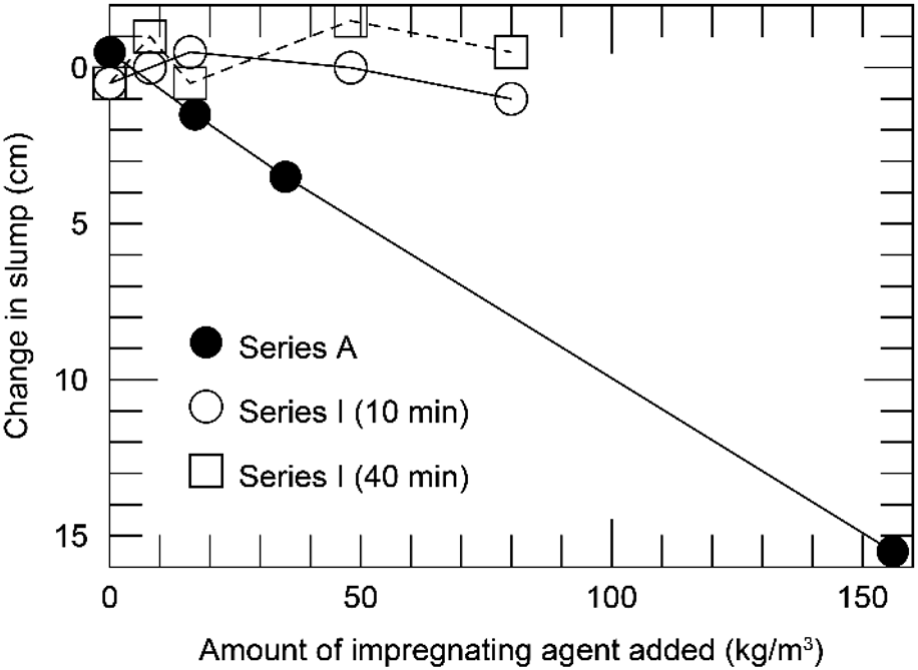
Slump test results obtained immediately after the mixing of the impregnation material.

Figure 5 shows the effect of the time between mixing the impregnation material and conducting the slump test on the slump. The vertical axis represents the difference between the slump value tested immediately after mixing the impregnation material and the slump value of the concrete assessed 10 or 40 min after mixing the impregnation material. The positive values on the vertical axis indicate that the slump values obtained 10 or 40 min after mixing the impregnation material were lower than those obtained immediately after mixing the impregnation material, indicating a decrease in fluidity. Note that the vertical axis is positive downward.

The results for Series A in Fig. 5 show that the difference increased almost proportionally with the mixing amount of the impregnation material. As shown in Fig. 4, when the mixing amount of the impregnation material in Series A was 35 kg/m^3^ or higher, the slump decreased significantly because of the CSH gel generated immediately after the mixing. It is inferred that with increase in the amount of impregnation material mixed in, the amount of CSH gel generated increases over time, resulting in a decreased slump amount.

The results for Series I in Fig. 4 show that the difference in slump owing to the amount of addition of the impregnation material is not large; that is, it is approximately 25 cm in each case. A significant decrease in slump was observed in Series A when the mixing amount was 35 kg/m^3^ or more; however, when the mixing amount was greater than 48 kg/m^3^ and 80 kg/m^3^, the results were not significantly different from those for smaller mixing amounts.

Next, regarding the effect of the timing of the test on the slump, Series I slump tests were conducted three times: immediately after mixing the impregnation material, 10 min after mixing, and 40 min after mixing. In Fig. 5, the differences between the results immediately after mixing and 10 min and 40 min after mixing are indicated by circles and squares, respectively. The results after 10 min and 40 min were nearly zero, regardless of the amount of impregnation material mixed. In comparison with the results of Series A, the effect of the decrease in fluidity owing to the reaction of the impregnation material was extremely small.

The decrease in fluidity observed in Series A was not observed in Series I because the experiments in Series I were conducted with higher fluid mix proportions, such as by using a high-performance AE water-reducing material. From the above, it can be inferred that a certain level of workability can be ensured with the Series I mix proportions by using the materials selected for this experiment. However, the results in Fig. 4 show that the slump value for Series I was approximately 25 cm, which is close to the upper limit of the measurement. If the slump value becomes too high, the height is maintained because of the interlocking of the aggregate, making it difficult to display the original fluidity. It is highly likely that the fluidity of the cast concrete in this experiment was not properly evaluated. Therefore, henceforth, we discuss the results of the slump flow test for Series I.

**Fig. 6:**
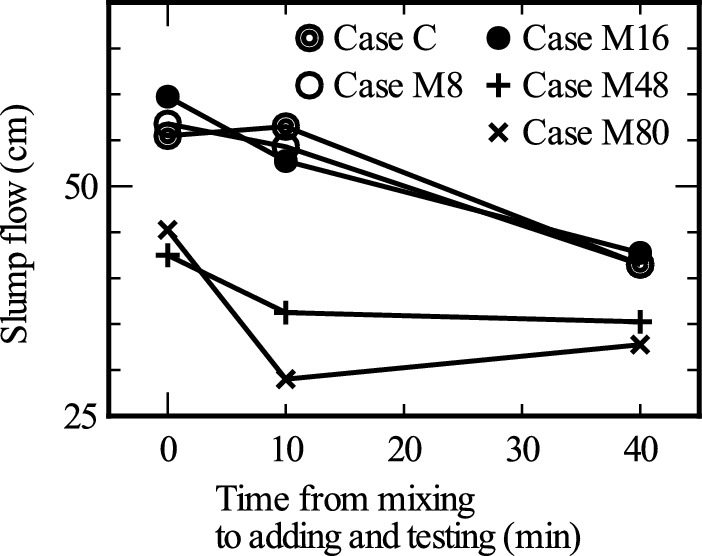
Slump test results obtained immediately after the mixing of the impregnation material.

Figure 6 shows the relationship between the slump flow values obtained in the slump flow tests and time interval between the mixing of the concrete and adding of the impregnation agent. First, the slump flow for Case M8, wherein 8 kg/m^3^ of the impregnation material was added, and that for Case M16, wherein 16 kg/m^3^ of the impregnation material was added, were almost the same as that in Case C, where no impregnation material was added. However, compared with these three cases, the slump flows for Cases M48 and M80, wherein 48 and 80 kg/m^3^ of the impregnation material were added, respectively, were approximately 2/3 to 1/2. These results confirm that the slump flow does not change if the mixing amount of the impregnation material is below a certain level but decreases once it exceeds a certain value.

Regarding the effect of the time between the mixing of the impregnation material and measurement of the slump, the slump flow decreased as the time until the test increased. The same was true for Case C, in which no impregnation material was added. The extent of decrease in the slump flow in Cases C, M8, and M16 were almost the same, and there seemed to be no effect of mixing the impregnation material.

### Compressive strength test

The compressive strength test results are shown in (Table 4).

**Table 4:** Compressive strength test results.

Label	Specimen age [days]	Case							
		C	M8	M16	M48	M80	M16-W45	M16-W50	M16-W60
(a) Series I									
Compressive strength [N/mm^2^]	7	19.5 (0.06)	18.5 (0.12)	20.2 (0.05)	7.34 (0.03)	1.86 (0.05)	-	-	-
	14	22.5 (0.15)	20.8 (0.07)	28.3 (0.04)	11.5 (0.08)	2.71 (0.11)	35.7 (0.01)	23.8 (0.16)	22.2 (0.09)
	21	26.2 (0.16)	23.4 (0.03)	27.5 (0.16)	13.2 (0.02)	3.63 (0.10)	35.1 (0.11)	32.0 (0.16)	20.5 (0.19)
	28	27.7 (0.09)	23.6 (0.04)	34.4 (0.06)	11.3 (0.06)	2.91 (0.21)	35.9 (0.10)	31.1 (0.11)	22.7 (0.17)
	49	-	-	-	16.1 (0.27)	-	-	-	-
	84	-	-	38.3 (0.03)	-	-	39.1 (0.06)	43.5 (0.03)	32.7 (0.06)
Young’s modulus [kN/mm^2^]	28	8.6 (0.47)	5.1 (0.66)	-	3.2 (0.47)	0.9 (0.12)	4.9 (0.27)	-	3.9 (0.31)

Label	Specimen age [days]	Case							
		C ‘	M’8-m0	M’16-m0	M’16–m15	M’16–m30	M’16–m60	M’48–m0	M’80–m0
(b) Series II									
Compressive strength [N/mm^2^]	28	15.6 (0.01)	12.5 (0.03)	8.89 (0.12)	12.9 (0.09)	11.8 (0.14)	12.0 (0.05)	5.25 (0.05)	1.16 (0.64)
Young’s modulus [kN/mm^2^]	28	5.3 (0.12)	2.9 (0.25)	1.7 (0.11)	3.4 (0.47)	2.2 (0.37)	2.6 (0.29)	1.2 (0.33)	-

*The numbers in the () are coefficients of variation.

#### Compressive strength

**Fig. 7:**
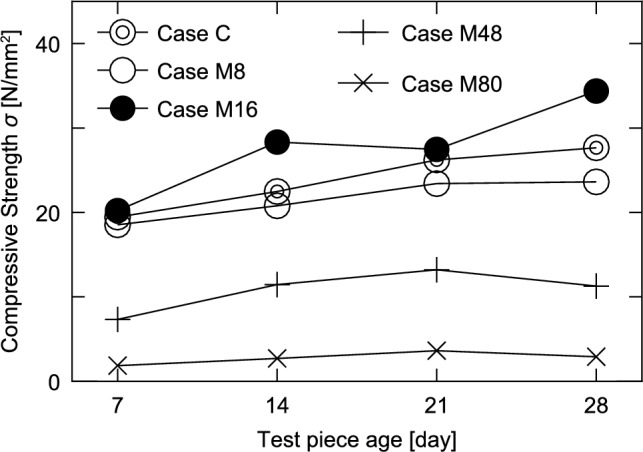
Relationship between concrete age of the specimen and compressive strength (Series I).

Figure 7 shows the relationship between the compressive strength (vertical axis) and specimen age (horizontal axis) for the case with W/C = 56% in Series I.

In Case C, where no impregnation material was mixed, the compressive strength increased from a low concrete age to the 28th day, indicating that the hydration reaction was progressing. In contrast, in Case M48 utilizing 48 kg/m^3^ of the impregnation material and Case M80 utilizing 80 kg/m^3^ of the impregnation material, the compressive strength decreased slightly from the 21st to the 28th day. In addition, the compressive strength of Case M48 was approximately one-third of that of Case C, whereas that of Case M80 was even lower than that of Case M48. In these cases, it was inferred that the hydration reaction was inhibited by the addition of the impregnation material, preventing the development of strength. In addition, the reaction of the impregnation material caused the voids to become larger, making it impossible to build a structure that could support the load.

However, for Case M8, in which 8 kg/m^3^ of the impregnation material was mixed, the strength continued to increase from a low concrete age to the 28th day, as in Case C. Although the developed strength was lower than that of Case C, the difference from the value in Case C was smaller than those of Cases M48 and M80. From the above, it can be said that as the mixing amount increases, it has a stronger effect of inhibiting the hydration reaction, resulting in a significant decrease in the compressive strength. However, in this experiment, with a mixing amount of 8 kg/m^3^, the inhibiting effect of the impregnation material on the hydration reaction was limited, and no significant decrease in strength occurred.

Case M16, which had a mixing amount of 16 kg/m^3^, achieved a greater strength than that of Case C. This is attributed to the CSH gel produced from the reaction of the impregnation material. The production of the CSH gel in the voids in the concrete densifies the entire concrete. However, this reasoning cannot be applied to Case M16, as explained below.

**Fig. 8:**
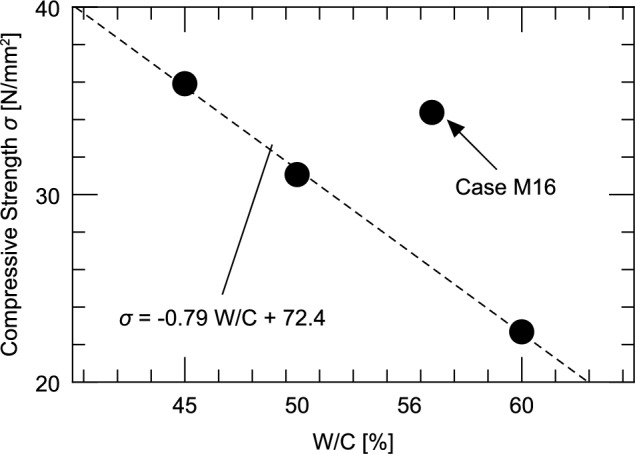
Relationship between water-cement ratio and compressive strength.

Figure 8 shows the 28 day compressive strength of four Series I cases, produced by the mixing method, with the amount of impregnation material set at 16 kg/m^3^ and the W/C ratio varied. This figure shows that the highest compressive strength is achieved in Case M16-W45 with a W/C of 45%, and the lowest in Case M16-W60 with a W/C of 60%. The compressive strength of Case M16- W50 with a W/C of 50% is between the values in the above two cases.

Furthermore, the compressive strength in Case M16 with a W/C of 56% is higher than that in Case M16-W50 with a W/C of 50%, and is approximately the same as that in Case M16-W45 with a W/C of 45%. The compressive strength development in the mixing method is attributed to the hydration reaction of cement and reaction of the impregnation material. In both cases, calcium components are consumed; hence, we infer that the greater the proportion of cement, the greater is the strength.

**Fig. 9:**
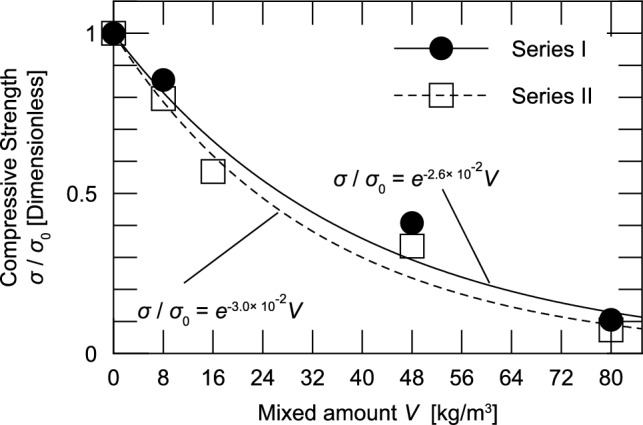
Effect of amount of impregnation material mixed on the compressive strength.

Figure 8 shows the fitting results for the three cases, excluding Case M16. As with normal concrete, the relationship between the compressive strength and water–cement ratio in the cases that employed the mixing method can be modeled using a linear function s = a W/C + b (a =  − 0.79, b = 72.4). However, only Case M16 deviates significantly from the correlation equation. In addition, a comparison between the results of Series II, which will be described later in Fig. 9, and the results of^9,18,19^ mentioned in Section “Introduction” shows a decreasing tendency of the compressive strength as the mixing amount increases, whereas the results of Case M16 show a different trend.

Because the reaction in the mixing method has not been fully elucidated, the possibility that the strength increases significantly because of the abovementioned reasons cannot be ruled out; however, considering the results of Figs. 7, 8, it is highly likely that the experiment for Case M16 was not performed correctly. Thus, Case M16 is excluded from the following discussion.

From Fig. 8, the relationship between the compressive strength and water–cement ratio can be expressed as Eq. (2).

2 $$\sigma_{1} = - 0.{\text{79 W}}/{\text{C}} + {72}.{4},$$

where ${\sigma }_{1}$ is the 28 day compressive strength [N/mm^2^] of concrete produced by adding 16 kg/m^3^ of the impregnation material immediately after preparation and measured at 28 days after mixing, and W/C is the water–cement ratio [%].

As in the case of normal concrete, we confirmed that the compressive strength tended to decrease as the W/C increased. The 28 day compressive strength of Case C (W/C = 56%), which did not contain any impregnation material, was less than 30 N/mm^2^, as shown in Fig. 7). This value was slightly lower than that of Case M16-W50. In this experiment, by increasing the W/C by 6%, compressive strengths equivalent to those of the concrete containing no impregnation material were obtained.

Figure 9 shows the relationship between the amount of impregnation material mixed and compressive strength from the results of Series I and II. All the results were non-dimensionalized for the cases with no mixing of the impregnation material (Case C for Series I and Case C’ for Series II). The compressive strength was measured at a concrete age of 28 d.

Figure 9 shows that the compressive strength decreased as the amount of impregnation material increased, as also seen in Fig. 7. This downward trend was modeled using an exponential function for both series. It is interesting to note that the exponent values are nearly the same, − 2.6 for Series I and − 3.0 for Series II. By approximating the results for Series I and II, Eq. (3) can be obtained to represent the relationship between the amount of impregnation material and compressive strength of the specimens in which the impregnation material was added immediately after mixing the concrete with W/C = 56%.

3 $$\sigma = \sigma _{{0~}} e^{{ - 2.8V}} ,$$

where $\sigma$ is the 28 day compressive strength of concrete with W/C = 56% and impregnation material added immediately after mixing the concrete [N/mm^2^]; $\sigma$_0_ is the compressive strength of concrete without the addition of the impregnation material (W/C = 56%) [N/mm^2^]; and *V* is the amount of impregnation material mixed [kg/m^3^].

**Fig. 10:**
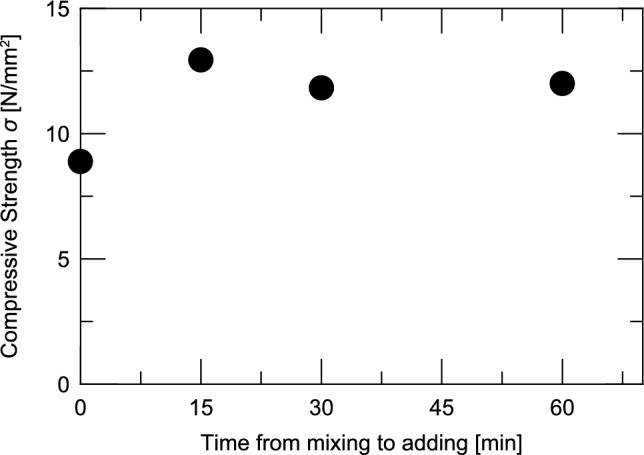
Difference in compressive strength depending on the time of addition of the impregnation material.

Next, we confirmed the effect of the mixing time of the impregnation material on the compressive strength. Figure 10 shows the relationship between the compressive strengths of Case M’16-m0, Case M’16-m15, Case M’16-m30, and Case M’16-m60 in Series II and the time from the start of mixing to mixing of the impregnation material. The compressive strengths are the values at a concrete age of 28 days.

Among the four cases, the compressive strength was the smallest in Case M’16-m0, in which the impregnation material was incorporated immediately after the start of concrete preparation. The compressive strengths of Case M’16-m15, Case M’16-m30, and Case M’16-m60 were 12.95, 11.83, and 12.00 N/mm^2^, respectively, which were 1.25 to 1.36 times greater than that in Case M’16-m0. The average ratio of the values in these three cases to the value in Case M’16-m0 was approximately 1.30. The results of this experiment showed that the compressive strength was 1.30 times greater when the time of mixing the impregnation material was delayed by 15 min or more after mixing the concrete. This is inferred to be due to the inhibitory effect of the impregnation material on the hydration reaction; thus, the local deficiency of calcium ions necessary for the hydration reaction can be inhibited by allowing the hydration reaction to proceed to a certain extent under the condition where the reaction caused by the impregnation material does not occur.

#### Young’s modulus

**Fig.11:**
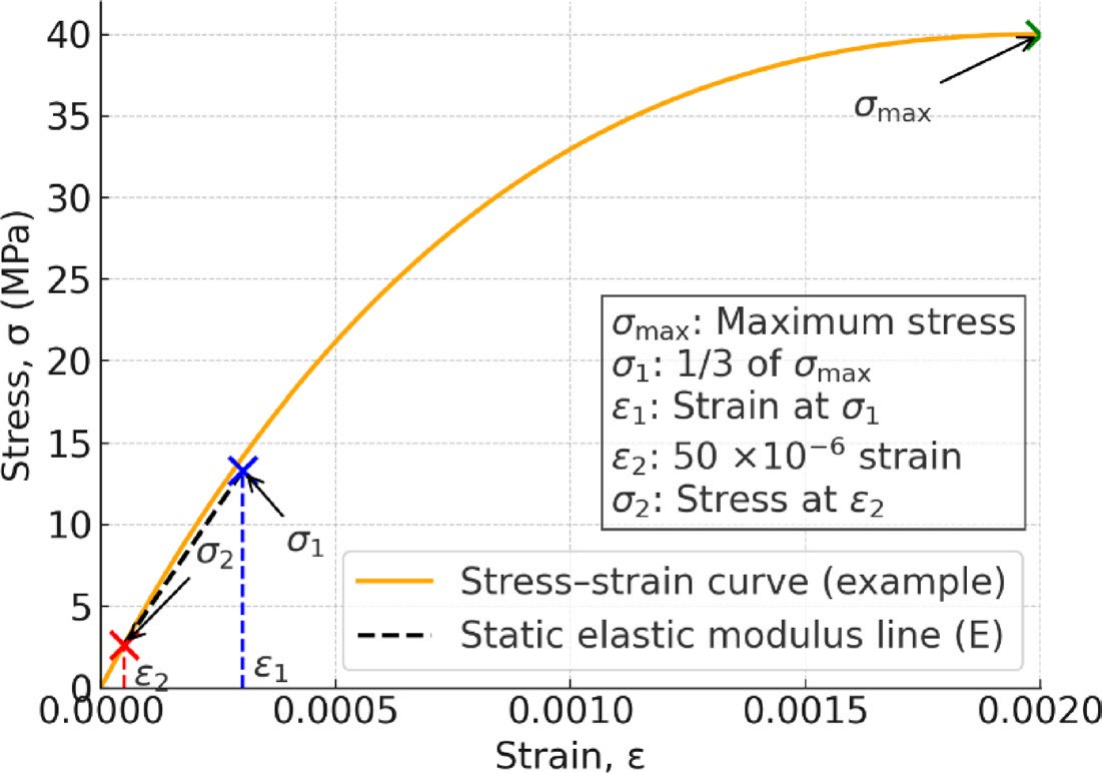
Static elastic modulus definition (JIS A 1149).

The static elastic modulus (Young’s modulus) was determined from the results of the compressive strength tests. According to JIS A 1149, the static elastic modulus is the gradient of the line segment connecting the stress point corresponding to 1/3 of the maximum load on the stress–strain curve of the specimen and the stress point when the longitudinal strain of the specimen is 50 × 10^−6^ (Fig. 11)^25^. It is calculated using the following equation.

4 $$E=\frac{{\sigma }_{1}-{\sigma }_{2}}{{\varepsilon }_{1}-{\varepsilon }_{2}}\times {10}^{-3},$$

where *E* is the Young’s modulus (kN/mm^2^), ${\sigma }_{1}$ is the stress at 1/3 of the maximum compressive stress (N/mm^2^), ${\sigma }_{2}$ is the stress when the strain is 50 × 10^–6^ (N/mm^2^), ${\varepsilon }_{1}$ is the strain at the occurrence of ${\sigma }_{1}$,

and ${\varepsilon }_{2}$ is the strain at the occurrence of ${\sigma }_{2}$. In these tests, the vertical displacement of the specimen used to calculate the strain was the displacement at the loading point.

**Fig. 12:**
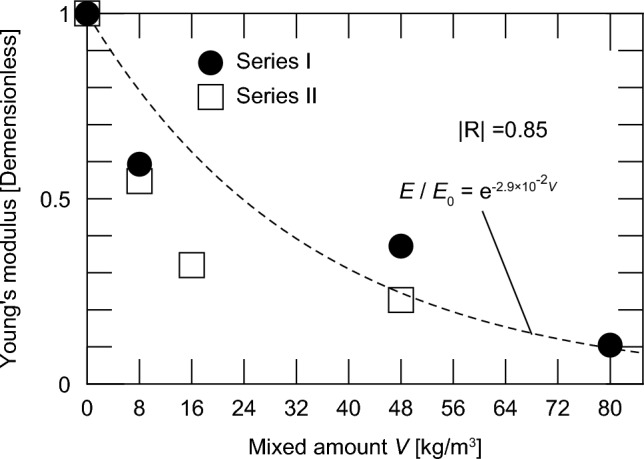
Relationship between the amount of impregnation material and Young’s modulus.

Figure 12 shows the relationship between the amount of impregnation material mixed and Young’s modulus at a concrete age of 28 days. As shown in Fig. 9, Series I is nondimensionalized in Case C, and Series II is nondimensionalized in Case C’.

Similar to the compressive strength shown in Fig. 9, the Young’s modulus decreased as the mixing amount increased. In addition, the correlation between the Young’s modulus and mixing amount can be evaluated using an exponential function, similar to that in the case of the compressive strength.

5 $$E = E_{0} e^{{ - 2.9 \times 10^{{ - 2}} V}},$$

where $E$ is the 28 day compressive strength of concrete with W/C = 56% and impregnation material added immediately after concrete preparation [N/mm^2^]; ${E}_{0}$ is the compressive strength of concrete without the impregnation material (W/C = 56%) [N/mm^2^]; and *V* is the amount of impregnation material mixed [kg/m^3^].

The value of the exponent is almost the same as that of the compressive strength. The correlation of the equation is not as consistent as that of the compressive strength, but it shows a relatively high value of -R- = 0.85.

**Fig. 13:**
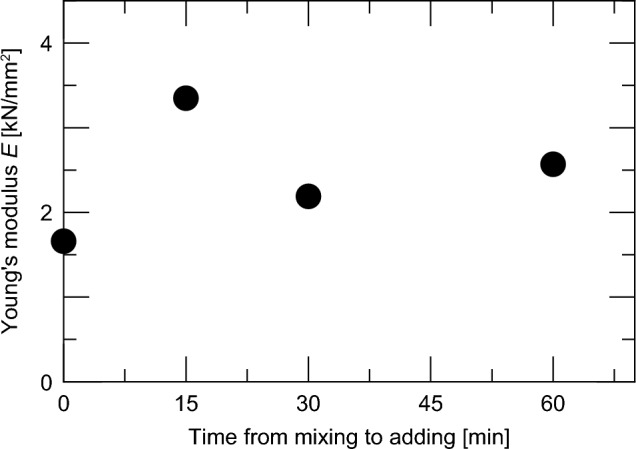
Effect of the time of mixing of the impregnation material on the Young’s modulus.

Figure 13 shows the relationship between the time of mixing of the impregnation material and Young’s modulus. As with the compressive strength in Fig. 11, there was a tendency for the Young’s modulus to improve when the mixing was delayed. However, as shown in Fig. 12, the variance was larger than that of the compressive strength. In this study, the strain was calculated from the displacement of the loading plate for experimental reasons, which may have resulted in large measurement errors.

The average Young’s modulus for the three cases in which the admixture was added 15 min or more after the concrete preparation began was 2.70 [kN/mm^2^]. Similar to the compressive strength, the ratio of the average value of these three cases to the Young’s modulus of the case where the admixture was added at the start of concrete preparation was 1.63. Although the Young’s modulus exhibited greater variability than the compressive strength, delaying the addition time by 15 min or more increased the Young’s modulus.

From the above, it was found that, as in the case of the compressive strength, although the Young’s modulus decreased with an increasing amount of impregnation material mixed, it was possible to inhibit the degree of decrease by delaying the time of mixing.

**Fig. 14:**
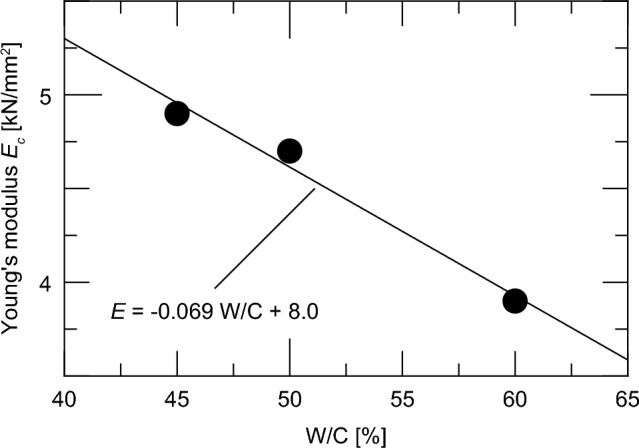
Relationship between water–cement ratio and Young’s modulus.

Finally, the relationship between the water–cement ratio and Young’s modulus was confirmed. Figure 14 shows the relationship between the Young’s modulus and water–cement ratio at 28 days of age. Similar to the compressive strength shown in Fig. 8, the two are linearly related, and their correlation can be expressed as the following equation:

6 $$E = - 0.069\frac{W}{C} + 8.0,$$

where $E$ is the Young’s modulus at 28 days [kN/mm^2^] and *W/C* is the water–cement ratio [%].

### Accelerated carbonation test

**Fig. 15:**
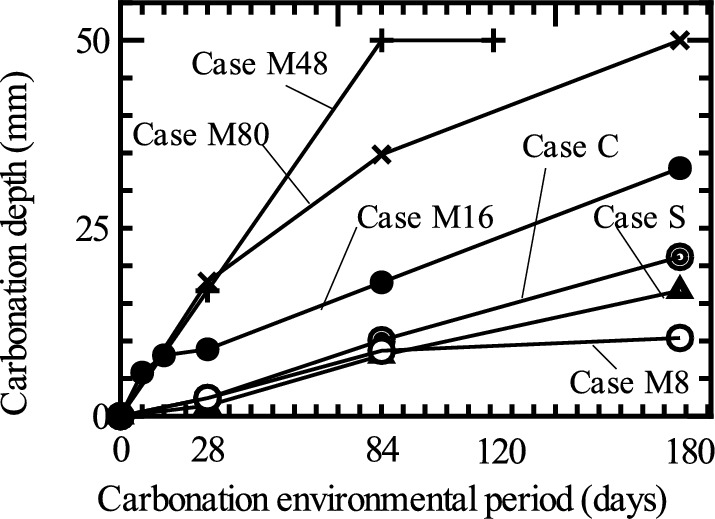
Time history of carbonation depth (W/C = 56%).

Table 5 lists the results of the carbonation tests. Figure 15 shows the time history of the carbonation depth measured for the experimental case with a W/C ratio of 56%.

**Table 5:** Measurement results of carbonation depth [mm].

Carbonation environmental period [days]	Case								
	C	S	M8	M16	M48	M80	M16–W45	M16–W50	M16–W60
7	–	–	–	5.8	–	–	0	2.5	3.6
14	–	–	–	8.1	–	–	0.3	6.5	8.1
28	2.4	1.4	2.4	8.9	16.7	17.9	1.5	8	9.4
84	10.1	8.1	8.7	17.8	50.0	34.8	11	12	16.7
120	–	–	–	–	50.0	–	–	–	–
180	21.2	16.7	10.4	33.0	–	50.0	20	21	35.5

The carbonation depths in the cases where the amount of impregnation material mixed was 16 kg/m^3^ or more (i.e., Case M16-W56, Case M48, and M80) were greater than that in Case C, where no impregnation material was mixed. This result indicates that the addition of the impregnation material accelerated carbonation. One possible reason for this is that the reaction of the impregnation material consumes the $\text{Ca}{\left(\text{OH}\right)}_{2}$ in the cement, causing a decrease in density. Note that in Case M48, no increase in the carbonation depth was observed after 84 d; this was not because carbonation was inhibited but because the entire surface of the test piece was neutralized and the alkaline parts disappeared (there was no longer any scope for carbonation to progress).

Upon examination of the results, Case C (no impregnation material was mixed), Case S (surface impregnation method was applied), and Case M8 (mixing method was applied with 8 kg/m^3^ of the impregnation material) did not show a significant difference in carbonation during the curing period from 28 to 84 days. However, at 180 days, the deepest carbonation was observed in Case C, followed by Case S. The measurement results of Case M8 on the 180th day did not differ significantly from that on the 84th day, indicating that carbonation was inhibited.

**Fig. 16:**
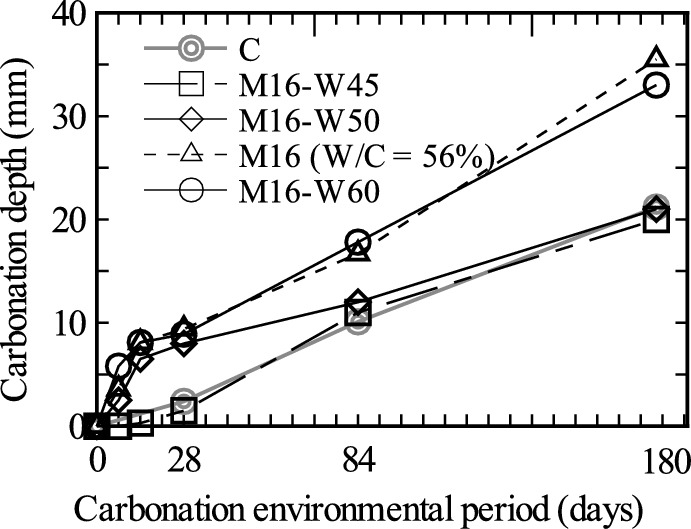
Effect of W/C on the change in carbonation depth over time.

Next, we examined the effect of the W/C ratio on carbonation inhibition. Figure 16 shows the time history of carbonation for the experimental cases M16-W45, M16, M16-W50, and M16-W60, where the amount of impregnation material was set at 16 kg/m^3^ and different W/C ratios were used. For reference, this figure also shows the results for Case C (W/C = 56%) in Fig. 3.

Among the mixing methods, carbonation was the most inhibited in Case M16-W45, which had the lowest water–cement ratio of 45%. The carbonation depth in Case M16-W45 was almost the same as that in Case C. In Case M16-W45, there was no significant change in the gradient from the start of the carbonation test to the 180th day, and it can be seen that the rate of increase in the carbonation depth was roughly constant. However, in Cases M16, M16-W50, and M16-W60, which had W/C ratios of 50, 56, and 60%, respectively, the carbonation depth increased rapidly immediately after the start of the acceleration test, but the rate of increase decreased after 14 days from the start of the carbonation test. In these cases, the initial reaction of the impregnation material did not proceed sufficiently because of the small amount of cement, and it is understood that the inhibitory effect of the impregnation material was exerted approximately 14 days after the start of the carbonation acceleration test.

In general, this experiment showed that as the W/C ratio decreased, the carbonation inhibition effect increased. This tendency is attributed to the fact that, as in the case of normal concrete, the tested concrete became denser with increasing proportion of cement, and the CSH gel produced by the reaction of the impregnation material further improved the density of the concrete.

### Summary

The findings from the experiments conducted in this study are summarized below.

#### Slump and slump flow tests

- Both slump and slump flow decreased with increasing dosage of the impregnating agent. This was attributed to pseudo-hardening caused by the formation of C–S–H gel through the reaction of the impregnating agent with water in fresh concrete.
- In this experiment, when a high-range AE water-reducing admixture was used and the dosage of the impregnating agent was limited to ≤ 16 kg/m^3^, slump and slump flow equivalent to those of conventional concrete were achieved. Under this condition, no reduction in slump or slump flow was observed even after 40 min from the start of mixing.

#### Compressive strength tests

- A significant linear relationship between compressive strength and the water-to-cement ratio (W/C) was observed, indicating that the strength–W/C law is applicable to the mixing method.
- Compared with conventional concrete, concrete produced by the mixing method exhibited lower compressive strength and Young’s modulus, with the extent of reduction increasing with higher dosages of the impregnating agent.
- The relationship between the degree of decrease in compressive strength and Young’s modulus and the amount of impregnating material added can be expressed by an exponential equation.
- Consistent with previous studies on sodium silicate mixing, the reduction in mechanical properties is considered to result from the consumption of calcium ions due to reactions of the impregnating agent in fresh concrete, which inhibited cement hydration.
- Based on this mechanism, it is inferred that allowing cement hydration to progress before adding the impregnating agent can mitigate reductions in mechanical properties. In this experiment, adding the impregnating agent 15 min after initiating cement–water hydration increased compressive strength by a factor of 1.36 and Young’s modulus by a factor of 1.63.

#### Accelerated carbonation tests

- The carbonation resistance was highly dependent on the dosage of the impregnating agent. In this experiment, a dosage of ≤ 8 kg/m^3^ achieved suppression effects comparable to the surface impregnation method. This is likely due to C–S–H gel formation by the reaction between silicates and cement hydration products, which filled and refined pores in the concrete. However, when the dosage exceeded 8 kg/m^3^, carbonation progressed more rapidly than in the non-incorporated control. This was presumably because the reaction of the impregnating agent inhibited the formation of hydration products, leading to reduced concrete density.
- The CO₂ suppression effect of the mixing method increased as the W/C ratio decreased, likely because lower W/C increased cement content and produced denser concrete.
- In this study, setting the impregnating agent dosage to 8 kg/m^3^ and W/C = 56% resulted in CO₂ suppression effects comparable to or greater than those of the surface impregnation method.

## Estimation formulas for compressive strength and Young’s modulus and confirmation of estimation accuracy

In this section, based on the relationship between the compressive strength and Young’s modulus shown in Sect.  5 and value of each parameter set as the experimental condition, we propose the equations for estimating the compressive strength and Young’s modulus under each condition and verify the estimation accuracy of the proposed equations.

### Estimation formulas

From Eqs. (2), (3) and the results in Fig. 10, the 28 day compressive strength of the concrete produced by the mixing method can be estimated using the following equation:

7 $$\sigma _{c} = \sigma _{{0~}} e^{{ - 2.8 \times 10^{{ - 2}} V}} \frac{{ - 0.79W/C~ + ~72.4}}{{28.1}}A,$$

where ${\sigma }_{c}$ is the calculated value of the 28 day compressive strength of concrete produced by the mixing method [N/mm^2^]. $\sigma$_0_ is the compressive strength of concrete without the impregnation material (W/C = 56%) [N/mm^2^], and *V* is the amount of impregnation material mixed [kg/m^3^]. ${\sigma }_{0}$ can also be referred to as the standard strength under the standard conditions: W/C = 56% and *V* = 0. *A* is a coefficient used to correct the time of mixing; it is 1.00 if the impregnation material is incorporated at the same time as the mixing of the concrete or if *V* = 0, and 1.30 if mixed after 15 min or later. The correction of the water–cement ratio was based on the result for W/C = 56%.

Equation (8) can be used to calculate the Young’s modulus at 28 days of age, using a water–cement ratio of 56% as the reference value.

8 $$E_{c} = E_{{0~}} e^{{ - 2.9 \times 10^{{ - 2}} V}} \frac{{ - 0.069W/C~ + 8.0}}{{4.1}}B,$$

where ${E}_{c}$ is the calculated value of the Young’s modulus of concrete produced by the mixing method [N/mm^2^]. ${E}_{0}$ can also be referred to as the standard Young’s modulus under the standard conditions of W/C = 56% and *V* = 0. *B* is a coefficient to correct the time of mixing; it is 1.00 if the impregnation material is incorporated at the same time as the mixing of the concrete or if *V* = 0, and 1.63 if it is mixed after 15 min or later. Note that the correction of the water–cement ratio is based on the result for W/C = 56%.

### Estimation accuracy

**Fig. 17:**
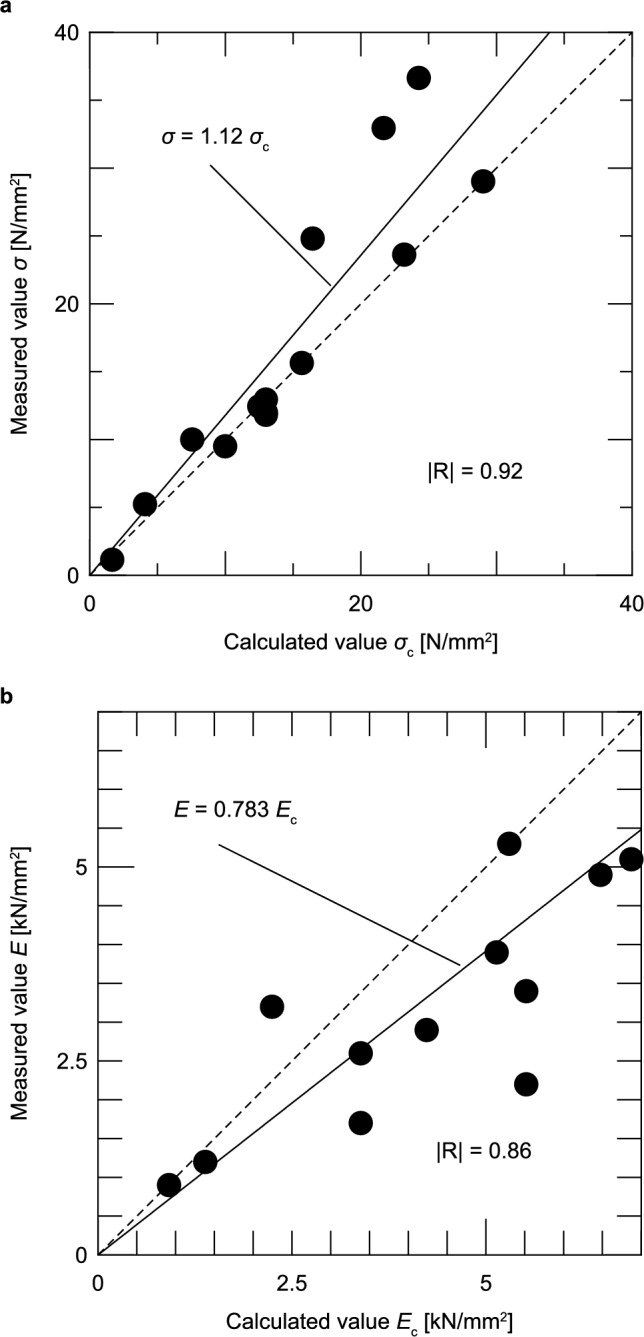
Comparison between measured and estimated values.

Figure 17 compares the estimated values calculated using Eqs. (7), (8) with the experimental values obtained for all the cases covered in this study. Figure 17a shows the results of the compressive strength and Fig. 17b shows the results of the Young’s modulus.

Figure 17a shows that although the estimated compressive strength tends to be lower than the measured values, the bias is 1.12, which is close to 1.0, and -R- is 0.92, indicating a relatively high value. Therefore, it can be considered that Eq. (7) can reasonably estimate the strength of the admixture method.

In the results of the Young’s modulus in Fig. 17b, the estimated values tend to be higher than the measured values. The bias is 0.783 and -R- is 0.86, both of which are smaller than the results for the compressive strength, indicating a lower estimation accuracy; this is attributed to the large measurement errors in the deformation of the test specimens, as mentioned in the previous section.

### Summary of estimation formulas and their accuracy

As described above, we proposed the equations for calculating the compressive strength and Young’s modulus at 28 days under various conditions (mixing ratio, mixing time, and water–cement ratio).

When comparing the calculated values obtained from the proposed equation with the measured values obtained from the experiments, the estimated compressive strength was found to be close to the measured values, demonstrating a high estimation accuracy.

However, it should be noted that the applicability of the proposed estimation formula is limited to the conditions confirmed in this experiment. The coefficients in the proposed formulas can be used under the experimental conditions described above as well as when the mixture design and materials listed in Table 1 are considered.

This study had the limitations that it did not explore a wide range of experimental cases and the proposed formulas have low generalizability. Nevertheless, it successfully demonstrated the usefulness of the proposed mixing method. Further experiments under various conditions are expected to be conducted in the future, leading to the development of more practical estimation formulas.

## Conclusion

This study investigated the incorporation of a silicate-based impregnating agent into fresh concrete, focusing on slump flow, CO₂ reduction potential, and compressive strength. The following conclusions can be drawn:

1. Excessive dosage induced pseudo-hardening and inhibited cement hydration, significantly reducing workability. However, with a high-range AE water-reducing admixture and a dosage of ≤ 16 kg/m^3^, workability equivalent to conventional concrete was maintained.
2. For carbonation resistance, excessive dosage decreased performance below that of the control, whereas limited dosage achieved results comparable to or exceeding surface impregnation, attributed to pore filling by C–S–H gel formation.
3. The impregnating agent reduced compressive strength and Young’s modulus, with greater reductions at higher dosages.
4. Lowering the water-to-cement ratio or delaying agent addition improved strength and stiffness.
5. An empirical equation was developed to estimate compressive strength and Young’s modulus from dosage, mixing time, and W/C ratio.

Overall, optimizing dosage and composition can achieve deterioration resistance equal to or greater than conventional methods without compromising workability or mechanical performance. The predictive equation offers a practical tool for performance estimation from mixture parameters. However, the equation’s accuracy and applicability remain limited by the available experimental data, and further studies are needed to expand its scope and improve predictive reliability.

## Data Availability

All data supporting the findings of this study are available within the article.
